# Survival of gastric cancer patients based on pathologic and demographic characteristics in Mazandaran between 2007 and 2013 

**Published:** 2019

**Authors:** Abolfazl Nikpour, Alireza khalilian, Iradj Maleki, Hossein Mohsenipouya, Jamshid Yazdani Charati

**Affiliations:** 1 *Student Research Committee, Faculty of Health, Mazandaran University of Medical Sciences, Sari, Iran *; 2 *Cancer Research Center, Mazandaran University of Medical Sciences, Sari, Iran *; 3 *Faculty of Nursing, Mazandaran University of Medical Sciences, Behshahr, Iran*; 4 *Department of Biostatistics, Health Sciences Research Center, Addiction Institute, Mazandaran University of Medical Sciences, Sari, Iran*

**Keywords:** Gastric cancer, Survival analysis, Kaplan Meier estimate

## Abstract

**Aim:**

Survey of the survival levels of gastric cancer and its effective causes.

**Background:**

The survival of gastric cancer because of the advances in this type of cancer cures has been increased during the last decades.

**Methods:**

643 patients evolved by gastric cancer referred to Imam Khomeini hospital of Sari (2007- 2013) were studied. According to this method, the numbers of 74 patients were neglected because of defective data, and the number of 569 patients went under study. The level of survival was determined by use of Kaplan Meier, so to determine the causes affecting on the patients' survival, the univariate analysis of Log-rank test was used.

**Results:**

Regarding the follow up of these patients during 2013 Nov-Dec the one, 2, 3, 4 and 5 years of survival of these patients were estimated equal to 0.77, 0.65, 0.52, 0.44, 0.27 percent and the survival median equal to 19 months, so survival means equal to 24.49 months. Based on the ranked logarithm test and FDR method some variables like stage (p<0.001) Radiotherapy (p<0.005) and undergo Surgery before Adjuvant chemotherapy (p<0.001) were determined as the effective factors on the survival probability.

**Conclusion:**

The life length of the patients under this article in comparison with developed countries is shallow that might be because of late reference or delayed diagnosis and the shortness of cure facilities. In this way, some materials like soon diagnosis and screen methods could be effective on the increase in patients' survival.

## Introduction

 Being one of the diverse types of cancers and one of the most important causes of death all over the world, gastric cancer is caused by uncontrolled growth of gastric cells (1). Cancer is the first ranked cause of death in developed countries, but the second in undeveloped (2). Gastric cancer is the second common type of diseases after lung cancer in men, however, breast cancer is the most common one in women (3). 

Half of the cancer cases occur in eastern Asia, mainly in China (4). Despite efforts done in the field of cancer prevention- occur, this disease has been spread increasingly and so is raised as a cause of death all over the world.

Because of the determination of danger causes and some controls, the level of death occur because of gastric cancer has been considerably decreased during the last 75 years. The level of death toll because of gastric cancer in the United States of America among men has been reduced from 28 to 5.8 per 100,000 people while among the women this census has been dropped from 27 toward 2.8 per 100,000 women (5). Despite this census, the number of 21260 new cases has been distinguished along the US, and 11210 diseased American has died because of this cancer. The level of cancer outbreak is still high in some countries like Japan, China, Chile, and Ireland (6).

Although in Iran, gastric cancer is ranked after road accidents and cardiovascular diseases at the third step of death causes and is raised as one of the most critical health and cure problems. Based on the estimations of the International Agency of Cancer Researches, during 2012, about 53,000 Iranian citizens have died because of cancer. The cancer of stomach systems are the most important types of cancer that according to medicals, some causes like environment, biologic, and diet cultures are engaged in its occurrence. Gastric cancer along Iran is ranked after breast cancer as the second common cancer in Iran with 11.4% of happening. The north and western north areas of Iran are the most dangerous areas noted by this type of cancer insomuch Mazandaran province (Northern Iran) has been reported as one of the most hazardous regions of Iran. Although it seems that the level of gastric cancer is going to be decreased because of its backdoor spirit, it is so hard to diagnose on timely so about the 50% of its diagnostic cases are on the advances stages (4, 7). Therefore, it is so important to have an accurate census of cancer occurrence and the death toll because of cancer to apply for some control programs in this country.

The purpose of this article was to study the survival and demographic- pathologic characteristics of the patients engaged by gastric cancer referring to the hospital of Imam Khomeini of Sari during 2007-2013. 

## Methods

Being a historical cohort study the statistical population of this article is consists of all patients involved by gastric cancer that have been referred to the Imam Khomeini hospital of Sari and have a cure record file in this hospital. The Clinical Research Ethics Committee approved this study of the Mazandaran University of Medical Science with code IR.MAZUMS.REC.1394.1440. A brief explanation of the survey was given to the patients who met the inclusion criteria, and informed consent was obtained from all the participants.

The understudy variables are three groups of demographic, biologic, and socio-economic variables (Table 1). The data was collected from the medical files of patients. The last statue of the patients’ health was determined by telephone contact and recording in the checklists. The survival time of the patients was calculated by subtraction the date of disease diagnoses (by endoscopy) from the date of death or censoring date based on its month. In the current study, the cases of censures include the live patients at the end of research and the lost cases during follow up, also for everybody that has died during the gastric study cancer considered as the cause of death. The use of SPSS software Ver.18 analyzed the data, and to calculate the cumulative survival the methods of cross-sectional statistical and Kaplan Meier were used. Therefore, for comparison between the levels of survivals the test of ranked logarithm was used and P<0.05 considered as the significant level. Because of some multiple similarities and so the error inflation type 1, the FDR (False Discovery Rate) was used as a significance test. 

**Table 2:** Factors affected on survival time of gastric cancer patients based on using COX regression

Variable	p-value	Rank	j/m)*0.05)		RH_0_
Family history	0.702	21	$\frac{21}{25}\times 0.05$	0.042	▀
Sex	0.723	22	$\frac{22}{25}\times 0.05$	0.044	▀
Age	0.191	12	$\frac{12}{25}\times 0.05$	0.024	▀
Job	0.232	13	$\frac{13}{25}\times 0.05$	0.026	▀
City	0.992	25	$\frac{25}{25}\times 0.05$	0.05	▀
Stage	<0.001	1	$\frac{1}{25}\times 0.05$	0.002	
site.of.tumor	0.741	23	$\frac{23}{25}\times 0.05$	0.046	▀
type.of.tumor	0.259	14	$\frac{14}{25}\times 0.05$	0.028	▀
metastasis.site.at.presentation	0.044	8	$\frac{8}{25}\times 0.05$	0.016	▀
OP.BEFOR	<0.001	2	$\frac{2}{25}\times 0.05$	0.004	
OP.AFTER	0.0613	9	$\frac{9}{25}\times 0.05$	0.018	▀
site.of.relaps	0.83	10	$\frac{10}{25}\times 0.05$	0.02	▀
time.of.relaps	0.038	7	$\frac{7}{25}\times 0.05$	0.014	▀
Progression	0.636	20	$\frac{20}{25}\times 0.05$	0.040	▀
time.of.prog	0.545	18	$\frac{18}{25}\times 0.05$	0.036	▀
type.of.regimen	0.019	5	$\frac{5}{25}\times 0.05$	0.010	▀
howmany.course	0.548	19	$\frac{19}{25}\times 0.05$	0.038	▀
CT	0.847	24	$\frac{24}{25}\times 0.05$	0.048	▀
Diarrhea	0.402	16	$\frac{16}{25}\times 0.05$	0.032	▀
Neuropathy	0.028	6	$\frac{6}{25}\times 0.05$	0.012	▀
Neutropenia	0.102	11	$\frac{11}{25}\times 0.05$	0.022	▀
cause.CT.dc	0.503	17	$\frac{17}{25}\times 0.05$	0.034	▀
RT	0.005	3	$\frac{3}{25}\times 0.05$	0.006	
TreatCompl	0.321	15	$\frac{15}{25}\times 0.05$	0.03	▀
reson.of.chemo.D.C	0.019	4	$\frac{4}{25}\times 0.05$	0.008	▀

**Figure 1:**
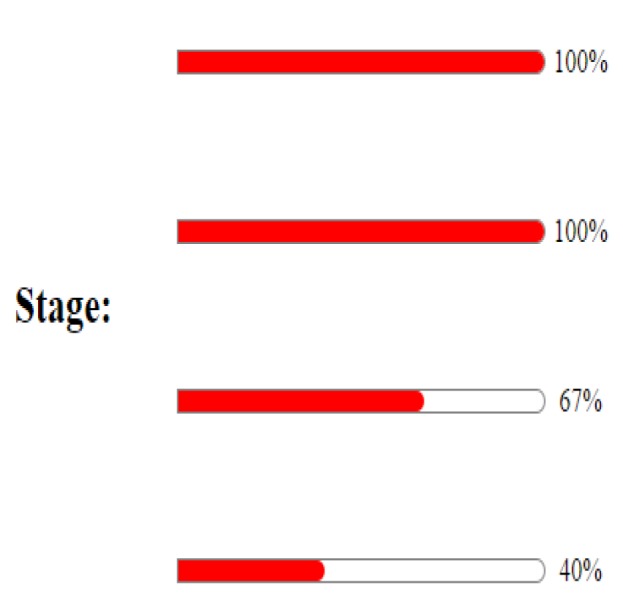
Survival diagram based on the disease stage

## Results

Among the 569 patients involved by gastric cancer under this study, the number of 381 patients male (70%), 163 patients women (30%) the average of age in the patients was calculated equal to 65.21 years. The average of the age of diagnosing in the involved men was equal to 65.68±13.41 years and in the women equal to 64.26± 14.49. Besides, 57.2% (318 patients) were among the patients with up to 65 years old and 9% (50 patients) among the patients with less to 45 years old age. Only two of the patients were unmarried. Among the 565 patients with recorded job information, the numbers of 154 men were working in the agriculture group, and 110 of women were working in a non-agricultural job (housewife). 60.8% of the patients were residing in urban and 30.2% in rural areas. For the number of 122 patients (40.1%) the tumor was located in the cardia and for 98 patients (32.2%) located in the Antrum. There was not seen any significant relationship between the time of survival and the location of the tumor. The data of the type of the tumor did exist in the medical file of the only 398 patients that among these patients 90.2% of them showed the tumor type of Adenocarcinoma. These 397 patients with the recorded stage of disease advance 315 of them (79.4%) were in the stage of the three and four at the time of referring to the hospital. Figure 1 shows survival diagram based on the disease stage.

**Table 1:** Demographic and pathologic characteristics of studied patients

Variable		N of death (%)	Total patients (%)
Sex	Male	224(72.5)	381(70)
	Female	85(27.5)	163(30)
City	Urban	170(61.2)	288(60.8)
	Rural	108(38.8)	186(29.2)
Job	Farmer	160(48.6)	222(39.3)
	Non farmer	169(51.4)	343(60.7)
Family history	Negative	114(56.2)	149(58.2)
	First degree GI cancer	50(24.6)	63(24.6)
	Second degree GI cancer	4(2)	5(2)
	First degree non GI cancer	33(16.3)	36(14.1)
	Second degree non GI cancer	2(1)	3(1.2)
Age	< 45 year	20(6.2)	50(9)
	45-65 year	114(35.3)	188(42.8)
	≥ 65 year	189(58.5)	318(57.2)
Stage	1	8(3.3)	27(6.8)
	2	27(11.3)	55(13.9)
	3	63(26.4)	98(24.7)
	4	141(59)	217(54.7)
Site.tumor	Cardia	66(39.3)	122(40.1)
	Body	21(12.5)	31(10.2)
	Antrum	48(28.6)	98(32.2)
	Lesser curvature	22(13.1)	36(11.8)
	Greater curvature	4(2.4)	6(2)
	Fundus	7(4.2)	11(3.6)
Type of tumor	Adenocarcinoma	211(91.7)	359(90.2)
	SCC+GIST+Adeno Signet Ring	19(8.3)	39(9.8)
Metastasissite at presentation	Liver	41(17.2)	72(17.6)
	Bone	25(10.5)	41(10)
	Liver+ Lung	3(1.3)	3(0.7)
	Para Aortic	6(2.5)	12(2.9)
	Lung	4(1.7)	5(1.2)
	Ascitis	8(3.3)	14(3.4)
	Paraourticlap+Liver	2(0.8)	5(1.2)
	Other	6(2.5)	9(2.2)
	No	144(60.3)	247(60.5)
Undergo Surgery before (Adjuvant Chemotherapy)	Yes	166(60.6)	294(63.8)
	No	108(39.4)	167(36.2)
Undergo Surgery after (Neoadjuvant Chemotherapy)	Yes	13(72.2)	32(78)
	No	5(27.8)	9(22)
Chemotherapy Receive or not	Yes	241(74.2)	425(76.4)
	No	84(25.8)	131(23.6)
Cause of DC of chemotherapy	Not indicated	5(6.3)	15(12.1)
	Low performance	6(7.5)	6(4.8)
	Refuse	5(6.3)	6(4.8)
	Consult	64(80)	97(78.2)
RT	Yes	81(27.5)	138(28.1)
	No	214(72.5)	353(71.9)

During this study, the number of 329 patients (57.8%) experienced the event (death), and for the 240 patients (52.2%) the censure event occurred (both alive at the end of the study and lost during the follow up).

Mean, median, standard deviation, of the patients' survival, was calculated equal to 24.49, 19, and 0.84 months. In the constant analysis using the method of Kaplan Meier and the levels of survival of 1, 2, 3, 4, 5 years for the patients were calculated 0.77, 0.65, 0.52, 0.44 and 0.27.

In order to comparison of the levels of survival in the different understudy subgroups the test of ranked logarithm was used and by use of FDR method it was determined that some variables like stage (p<0.001). Radiotherapy (p<0.005) and adjuvant Chemotherapy before undergoing surgery (p<0.001) had a significant relationship with survival ( Table 2).

## Discussion

Global statistics show that against the considerable decreasing trend of gastric cancer among the European countries like Spain (8) and Italy (9) in the lots of developing countries like Korea (10) Iran (11) and Portugal (12) it has an increasing trend. The trend of cancer occurrence in the west of Iran is increasing among both sexes male and female. 

In this research, no significant relationship between sexuality and the life length of the patients is observed. Kitagawa et al. lieu et al. and Jamali et al. showed this result and did not find any relationship between sexuality and life length of the patients (13-15). Based on the sexual distribution of the population, 381 cases (70%) of the patients were men and 163 cases (30%) were women that based on this data the ration of men to women is 3:2 and is coincident with the results of study done in Ardabil (16) and Fars (17) provinces. But based on the sexual ratios is more than some of the reviews and less than some others. In the current study, the age of diagnosis is not considered as one of the causes affecting on the patients' survival and is coincident with the results of Sirius et al. Xhang et al. and Ismaeeli et al. (18-20) but is in agreement with Speech et al Iunee et al. Roshanaee et al. Yazdanband and Moqimi Dehkordi et al (17, 21-24). Age mean of the patients in the current study was equal to 65.21 years (65.68 years for the men involved by gastric cancer and 64.26 years for the patient women that is more than mean estimations among the other studies (25, 33). In this study, the results showed that the most common contagion occurs in the seventh decade of the life that other studies confirm our findings (17, 25, 26). A similar study done at the University of Newcastle showed that most patients with gastric cancer are in the decade of 50-70 (27). The age difference among the patients based on the variables of location and situation of residency was not significant (like the cases observed in Iran). Despite this observation in another study done in France (28), the location of residence was indicated as a factor affecting patients' survival. In this study, the variable of the family background was not shown as an affective factor that is accordance with the results of Moqimi Dehkordi (17), Yazdanband (16) and Biglarian (4) but is not in accordance with the result done in other countries. 

Based on the current study the most common type of cancer is adonocarsinium and consists of 90.2% of the total population of studied persons, and the rate of contagion among the men reported more than women and is coincident with the studies done by Norouzinia et al. (29). This census confirms the censuses of Iran (30). The most common anatomic location for cancer is Cardia parts (40/1%) and then the Antrum part (32/25) that confirms the results shown by Dr. Sediqhi et al. (31). Also, Noroozinia (29) showed the Ednocarsinium as the first ranked part of cancer engagement and Cardia part as the second. The results Davoodabadi et al. shows the Antrum part as the central part of involvement(44%) but noted that during the last 2years ending to his study the cardia's participation has been increased considerably(32). Also, another research done by Dr. Taqhavi et al. shows that among the patients involved by gastric cancer with proportional abundance equal to 53.6 the most common involved part is Cardia( 18.9%) and then Antrum( 17.2%). The meant locations are based on the endoscopic Biopsy and can include cardia, antrum, and fondos. Also in the current study, the variable of existence or absence of metastasis was not shown significant against some of the previous studies that had shown an antagonistic relationship between metastasis life and patient’s survival (14, 23). The results of the current research is accordant with the results of Lieu et al. yoni et al. Noorikajoori et al (14, 22, 33)but is not accordant with the results of Ghorbanigholiabadi et al. Yazdani et al, Xhang et al. Jamali et al. Speech et al(15, 19, 21, 24, 34).

The results of Log-rank showed that there is a significant difference between the patients' longevity and the stage of cancer advances. The patients who had referred to the hospital at the 4th stage of the disease had less survival, and this result is accordant with all studies done in Iran and out of Iran country. 

In the current study the 1, 2, 3, 4 and 5 years of survival for the patients were calculated 65.77, 52.0, 44.0, 0.0 and 0.27 respectively but Youni et al. reported the 1 and 3 year survival of the patients with gastric cancer equal to 75% and 42% respectively(22). Xhang et al. during a study had reported the survival of 1, 3, and 5 years equal to 87%, 61% and 32% respectively (19). The results of the study done by Esmaeeli (20) in Mazandaran province (North of Iran) and by Yazdanbod (16) in Ardabil province (Western north of Iran) and Pourhoseingholi (35) are almost accordant with the current study but the results of studies done by Zera'ati (36) in Tehran (3 years survival: 0.31 and 5 years survival: 0.18) and Biglarian (4) in Tehran (3 years survival 0.32) are not accordant with the current study. The five years of survival for some developed countries like the United States, Switzerland, France, China, Japan had been reported 0.37, 0.22, 0.30, 0.30 and 0.35 respectively and the five years survival for another most of countries has been reported between 10- 30 % (37, 38).

In the current study, the method of treatment was not determined as a factor affecting the patients' survival. But in the studies done in Northern America (39) Sun in China (40) and in Europe (41) shows the positive effects of complementary chemotherapy and chemotherapy- radiotherapy on the patients' survival. 

Finally, this study shows that the survival of the patients involved with gastric cancer referring to the Touba Treatment Clinic Center of Sari is low that might be because of few facilities for on timed diagnoses and any strategic program to control these kinds of cancers in Mazandaran province. The ranking of the stages of cancer is the most crucial action to increase the survival of patients involved with gastric cancer. So the cause of lowness of the survival in these patients must be because of so late diagnosis and the advanced stage of their disease.
